# Highly efficient and simultaneous catalytic reduction of multiple toxic dyes and nitrophenols waste water using highly active bimetallic PdO–NiO nanocomposite

**DOI:** 10.1038/s41598-021-01989-7

**Published:** 2021-11-22

**Authors:** A. G. Ramu, Dongjin Choi

**Affiliations:** Department of Materials Science and Engineering, Hongik University, 2639-Sejong-ro, Jochiwon-eup, Sejong-city, 30016 Republic of Korea

**Keywords:** Ecology, Environmental social sciences, Natural hazards, Chemistry, Nanoscience and technology

## Abstract

Azo dyes and nitrophenols have been widely used in the various industry which are highly toxic and affecting the photosynthetic cycle of aquatic organism. The industry disposals increase the accumulation of azo compounds in the environment. In the present study, we synthesized the low cost, PdO-doped NiO hetero-mixture via simple hydrothermal combined calcination process. The morphology results proved that, the spherical PdO nanoparticles are evenly doped with NiO nanoparticles. The band gap values of metal oxides NiO, PdO and PdO–NiO composite were found to be 4.05 eV, 3.84 eV and 4.24 eV, respectively. The high optical bandgap (Eg) value for composite suggests that the PdO interface and NiO interface are closely combined in the composite. The catalytic activity of the PdO–NiO was analyzed for the reduction of different toxic azo compounds namely, 4-nitrophenol (NP), 2,4-dinitrophenol (DNP), 2,4,6-trinitrophenol (TNP), methylene blue (MB), rhodamine B (RhB) and methyl orange (MO) separately and their mixture with the presence of a NaBH_4_. For the first time, the large volume of the toxic azo compounds was reduced into non-toxic compounds with high reduction rate. The proposed PdO–NiO catalyst exhibit excellent rate constant 0.1667, 0.0997, 0.0686 min^−1^ for NP, DNP and TNT and 0.099, 0.0416 and 0.0896 min^−1^ for MB, RhB and MO dyes respectively which is higher rate constant than the previously reported catalysts. Mainly, PdO–NiO completes the reduction of mixture of azo compounds within 8 min. Further, PdO–NiO exhibit stable reduction rate of azo compounds over five cycles with no significant loss. Hence, the proposed low cost and high efficient PdO–NiO catalyst could be the promising catalyst for degradation of azo compounds.

## Introduction

Rapid industrial growth in the global era has caused environmental pollution problems. Aromatic nitrophenols (4-nitrophenol, 2,4-dinitrophenol, and 2,4,6-trinitrophenol) and azo dyes (Methylene blue, Rhodamine B, and Methyl orange) are the major contributors to the environmental contaminations^1–4^. The azo dyes are widely used in leather factories, metal plating factories, food companies, paint industries, pulp, especially in the textile industry^5^. However, during the dyeing process, only 5% of the dye is absorbed, and the rest of the dye is released into the environment. This process leads to environmental pollution and human health problems such as bleeding, nausea, and skin ulcers^6^. Another high-rated toxic organic contaminant is nitrophenol. This aromatic and inert compound is highly toxic and is emitted into the environment through wastewater from paper, pesticides, agrochemicals, dyes, and pharmaceutical industries^7,8^. Human exposure to these toxic nitrophenols adversely affects kidney and liver function and causes cancer to the living organism^9^. Even though the short-term exposure also causes severe health diseases to human beings such as nausea, cyanosis, and drowsiness^10^. Thus the U.S EPA listed the nitrophenols are top-rated toxic pollutants to the human and living organism^11^. Hence, the removal of these toxic azo compounds from the environment is a requirement. So far, degradation achieved by ultrafiltration, aerobic or anaerobic treatment, adsorption, advanced oxidation process (AOP), and coagulation techniques widely been employed to remove these nitrophenols and azo dyes^12,13^. However, the techniques mentioned above are said to be more expensive and less effective. On the other hand, catalytic reduction is an emerging process to the reduction of nitrophenols and azo dyes where the organic compounds are reduced to hydrogenated forms, which are non-toxic and eco-friendly products. Especially the reduction product of the nitrophenol, i.e., aminophenol (AP), is a crucial intermediate in the production of agrochemicals, pharmaceuticals, and corrosive coating industries.

However, the reduction of nitrophenols and azo dyes by BH_4_^−^ is catalyzed by many noble metals such as Pt, Au, Ag, and Ru^14–17^. However, the scarcity and high cost of the catalyst hinder practical development. As a result, the size- and shape-controlled synthesis of the catalyst has recently sparked enormous research efforts, which have led to the fabrication of various morphologies such as wires, rods, bristles, plates, polyhedrons, and branched nanostructures. Therefore, the solubility of all these metals should be recorded by one with similarly high catalytic activity. Also, a recent study by Mahmoud et al.^18^ provided clear evidence that the reaction continued at the surface.

In particular, bimetallic nanocrystals are a very attractive combination of noble metals and non-noble metals (cheap first-order conversion metals), which offers opportunities to reduce noble metal use and the overall cost of the catalysts. For example, Toshima et al.^19^ The hydrogenation of nitrobenzene by bimetallic Pd−Ni NPs were more effective than Pd NPs. Structures of bimetallic nanomaterials have aroused interest in their potential applications in organic reactions, fuel cells, and sensing devices. Accordingly, several synthetic approaches such as water heating systems, electrochemical deposition, seed-mediated growth, and galvanic conversion have been developed. In this present study, we have developed bimetal Pd–Ni-containing oxide by the facile hydrothermal preparation approach. The synthesized sample is characterized by the specialization of essential test techniques to identify their essential physical and chemical characters. The morphological results show that the spherical PdO nanoparticles are evenly doped with NiO. Catalytic reduction of azo dyes and aromatic nitrophenols using NaBH_4_ was carried out to evaluate the catalytic efficiency of the resulting catalyst. For the first time, we have demonstrated the reduction of azo compounds with higher volume than the traditional reduction process. The PdO doped NiO show efficient catalytic reduction of azo compounds than the pure NiO. PdO–NiO catalyst exhibit excellent rate constant for the reduction of azo compounds, which is higher than the reported other precious metal catalysts. Our findings give certain insight for the catalytic reduction based on the oxygen-containing transition bi-metal catalysts.

## Results and discussion

**Figure 1:**
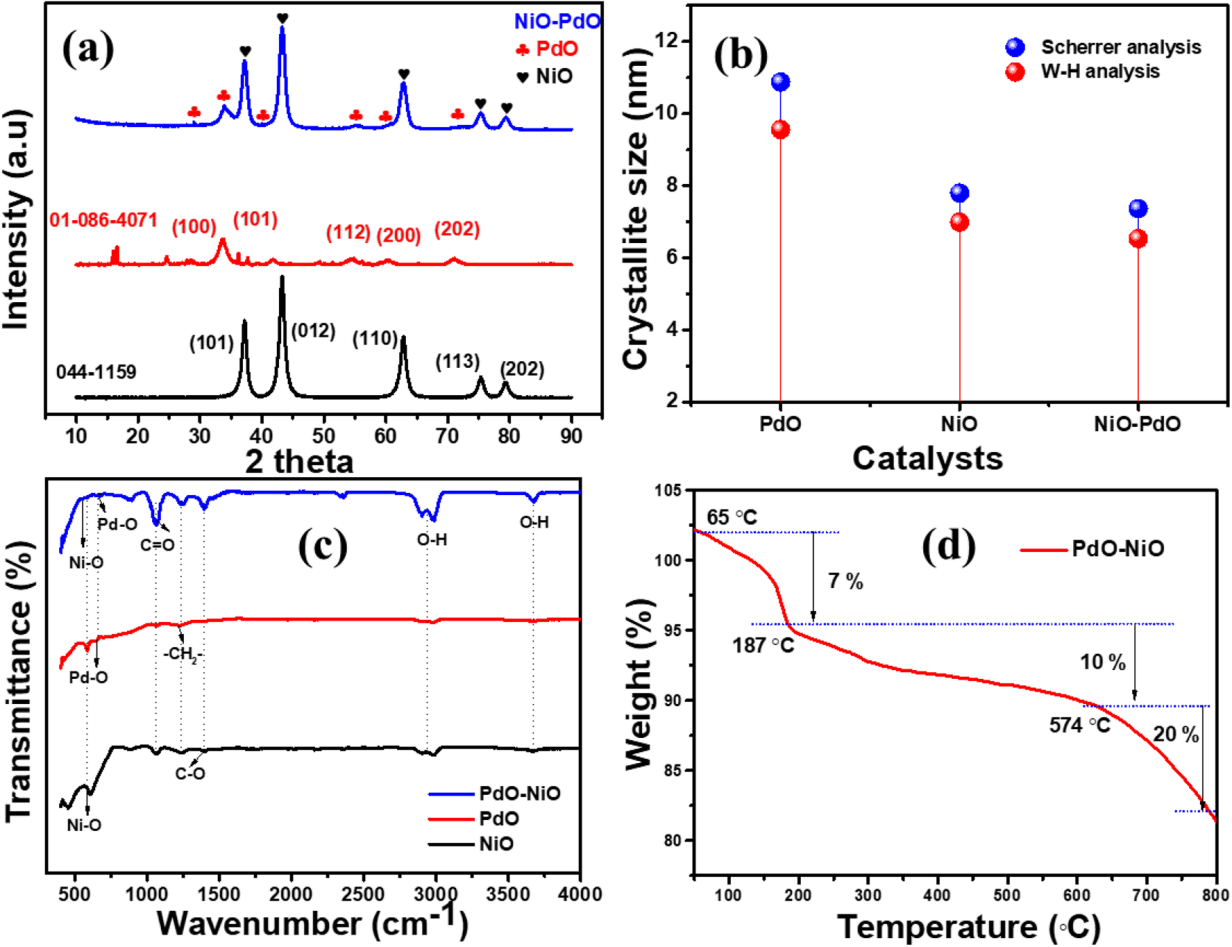
(**a**) X-ray diffraction pattern and (**b**) FTIR spectra of NiO, PdO and PdO–NiO (**c**) TGA curve of PdO–NiO nanocomposite.

The crystalline nature and phase purity of the synthesized NiO, PdO and PdO–NiO nanocomposite were confirmed by XRD analysis and shown in Fig. 1a. All three XRD patterns show the highly crystalline nature, which confirms the purity of the samples. In PdO–NiO mixed metal oxide, The intense diffraction peaks at 2θ = 37.16, 43.24, 62.81, 75.32 and 79.34° indexed to (101), (012), (110), (113) and (202) planes of the cubic phase of NiO, which is highly consistent with standard JCPDS NO: 01-071-4751^20^. On the other hand, the sharp peaks at 2θ = 34.54 and 55.79° indexed to (101) and (112) planes corresponding to the tetragonal crystalline phase of PdO with an average lattice parameter 3.043 Å, which is highly associated with standard JCPDS NO: 043-1024^21^. Interestingly, metallic diffraction peaks and other impurity phases were not observed in the hybrid PdO–NiO nanocomposite. This confirming that metal source (Pd) completely oxidized to the metal oxide (PdO) and formed hybrid PdO–NiO nanocomposite. In addition, the single metal oxide (PdO, NiO) diffraction patterns were compared in Fig. 1a.

The sharp peaks of each metal oxide confirm the high crystallinity of the tetragonal phase of the PdO and cubic phase of NiO. Both diffraction pattern of the single metal oxides is well associated with standard JCPDS file: 01-071-4751 and 043-1024 respectively. The average crystallite size, micro-strains of the NiO, PdO and PdO doped NiO were calculated by using Scherrer analysis and W–H analysis and plotted in Fig. 1b. A small variation was observed in the crystallite size of the catalysts (PdO, NiO and PdO–NiO) in Fig. 1b, which is due to the difference in the distribution of the crystal in the catalysts. The average crystallite size of the PdO, NiO and PdO–NiO was found to be 10.8, 7.8 and 7.36 nm, respectively. The PdO doping reduces the crystallite size in the PdO–NiO composite, consistent with previous reports. The calculated crystallite sizes, d-spacing, micro-strains, and binding energies of the PdO, NiO, and PdO–NiO are shown in the Table 1. In addition, the experimentally calculated d-spacing value of the pure PdO and NiO was well correlated with theoretical values and shown in Table SI. 1. The synthesized catalyst showed the negative and positive slopes of ε are corresponds to the compressive and tensile stress, respectively.

**Table 1:** Crystallite sizes, d-spacing, micro-strains, and binding energies of the PdO, NiO, and PdO–NiO.

Materials	Crystallite size D (nm)		Micro-strain ε	Average lattice (nm)	E_g (eV)_
	Scherrer	W–H			
PdO	11.9773	9.5586	2.27 × 10^–3^	0.240487	3.84
NiO	7.7957	6.5315	− 1.70 × 10^–3^	0.168989	4.05
PdO–NiO	7.3613	2.9967	− 1.32 × 10^–2^	0.184324	4.24

Furthermore, the chemical bonding and functional groups were analyzed by FTIR spectrometer. Figure 1c show FTIR spectra of the synthesized NiO, PdO and PdO–NiO nanocomposite. Similar spectra were observed for the three catalyst, the absorption peaks at high-frequency region 3200–3400 cm^−1^ belongs to O–H stretching vibration of the water molecules, due to surface adsorption phenomenon. Furthermore, three absorption peaks appeared at 1398.2, 1237.8 and 1057.8 cm^−1^, which ascribed to the C–O, CH_2_ and C=O stretching vibrations, which is well associated with XPS analysis data. The metal oxide bonding peaks appeared in the frequency range of 480.2–702.6 cm^−1^. Hence the Pd–O and Ni–O stretching frequency in the PdO doped NiO sample confirmed the formation of hybrid PdO–NiO nanocomposite^22^. After that, the thermal stability of the PdO–NiO nanocomposite was studied by TGA analysis. Figure 1d shows the thermogram of the PdO–NiO nanocomposite. The first weight loss (7%) started in the temperature range of 65 to 180 °C. Due to the H2O molecules, evaporation and then the sustainable weight loss of around 10% was observed in the range of 187 to 574 °C. Beyond 600 °C a significant weight loss 20% was observed, which may be assigned to the unreacted CO_3_ combustion^23^. Hence, the XRD, FTIR and TGA spectral studies confirmed the formation of hybrid PdO–NiO nanocomposite.

**Figure 2:**
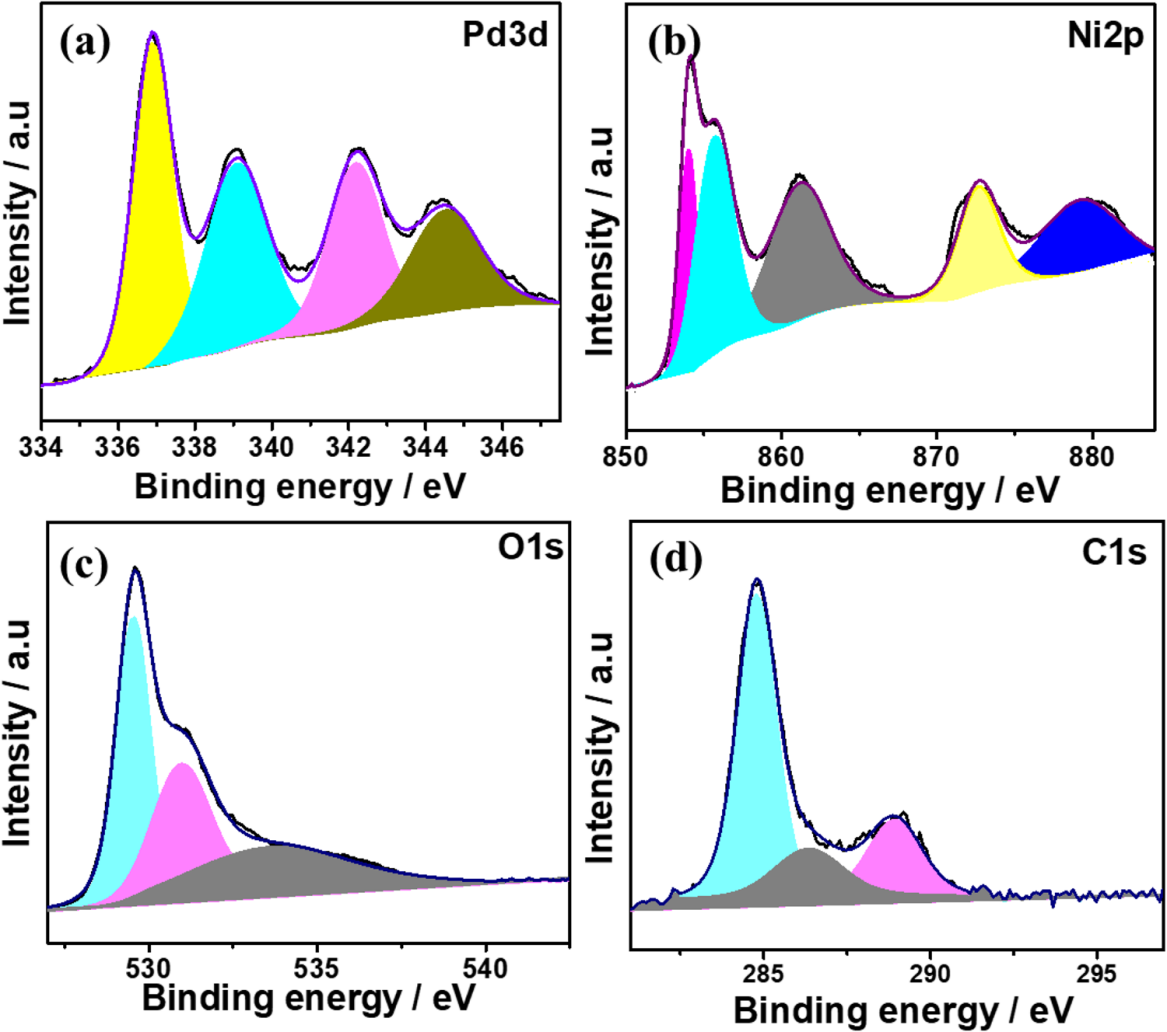
High resolution X-ray photoelectron spectroscopy (**a**) Pd 3*d*, (**b**) Ni 2*p*, (**c**) O 1*s* and (**d**) C 1*s* spectra of PdO–NiO nanocomposite.

The electronic state and chemical bonding of the PdO doped NiO composite was analyzed by using XPS spectra. Figure 2 shows the XPS spectra of the PdO–NiO nanocomposite, the broad scan spectrum (Fig. SI. 1) of the PdO–NiO, which show the existence of the Pd (3*d*), Ni (2*p*), O (1*s*) and C(1*s*) elements. The deconvoluted Pd 3d XPS spectrum in Fig. 2a shows two major peaks at a binding energy of 336.4 and 342.2 eV corresponds to spin–orbit doublets of Pd 3*d*^5/2^ and Pd 3*d*^3/2^, respectively, which confirmed Pd^2+^ ions in the form of PdO in the PdO–NiO nanocomposite^24,25^. In addition, the satellite peak of Pd species appeared at a binding energy of 339.2 eV and 345.3 eV. In Ni 2*p* spectra (Fig. 2b), Ni 2*p*^3/2^ and Ni 2*p*^3/2^ spin–orbit doublets peaks were observed at 854.9 eV and 872.5 eV, which corresponded to Ni–O and Ni–OH, respectively and their corresponding satellite peaks are located at a binding energy of 867.2 eV and 879.1 eV^26,27^. Furthermore, O 1*s* spectra (Fig. 2c) show the two peaks at a binding energy of 529.3 eV and 534.2 eV, which ascribed M–O and M–OH species. The obtained XPS spectra of the PdO–NiO nanocomposite are well associated with the XRD and EDX analysis.

**Figure 3:**
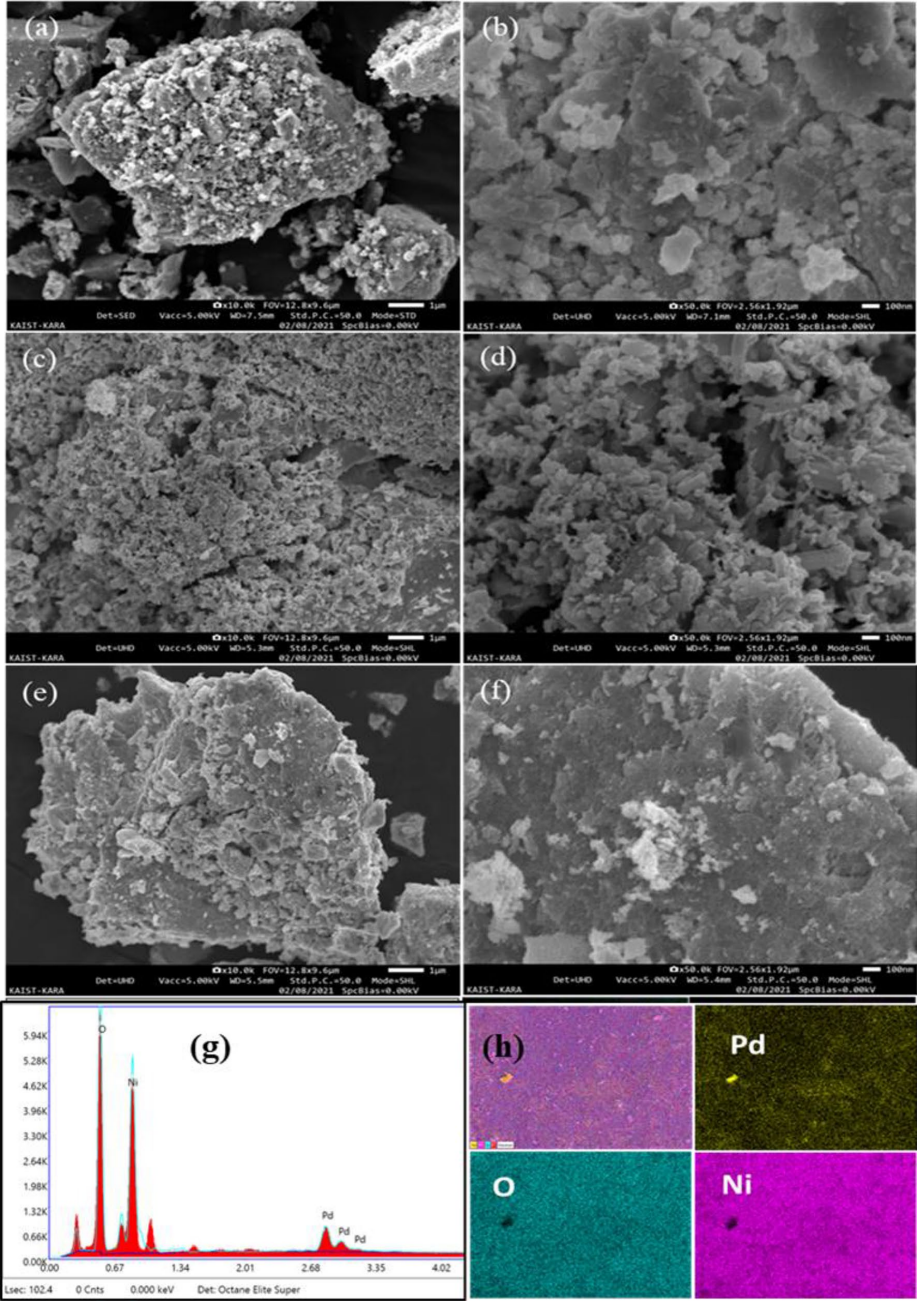
FESEM images (**a**,**b**) NiO (**c**,**d**) PdO, (**e**,**f**) PdO–NiO and (**g**,**f**) EDS spectra and elemental mapping of PdO–NiO nanocomposite.

The morphology feature and elemental composition of the prepared NiO, PdO and PdO–NiO nanocomposite was scrutinised by FE-SEM. Figure 3 shows the SEM morphology images of NiO, PdO and PdO–NiO nanocomposite at low and high magnification. Pure metal oxides NiO and PdO samples in Fig. 3a–d shows the porous crystalline morphology with high purity of the respective elements. On the other hand, PdO doped NiO sample in Fig. 3e,f shows the uniform, monodisperse, spherical crystalline morphology. Which confirms that PdO uniformly distributed with NiO. Hence, the PdO doping enhances the surface area of the catalyst. In addition, the elemental composition and elemental mapping was analysed for PdO–NiO sample and shown in Fig. 3g,h. The EDX spectra and elemental mapping clearly confirms the presence of the Pd, Ni and O elements in the composite with high purity.

**Figure 4:**
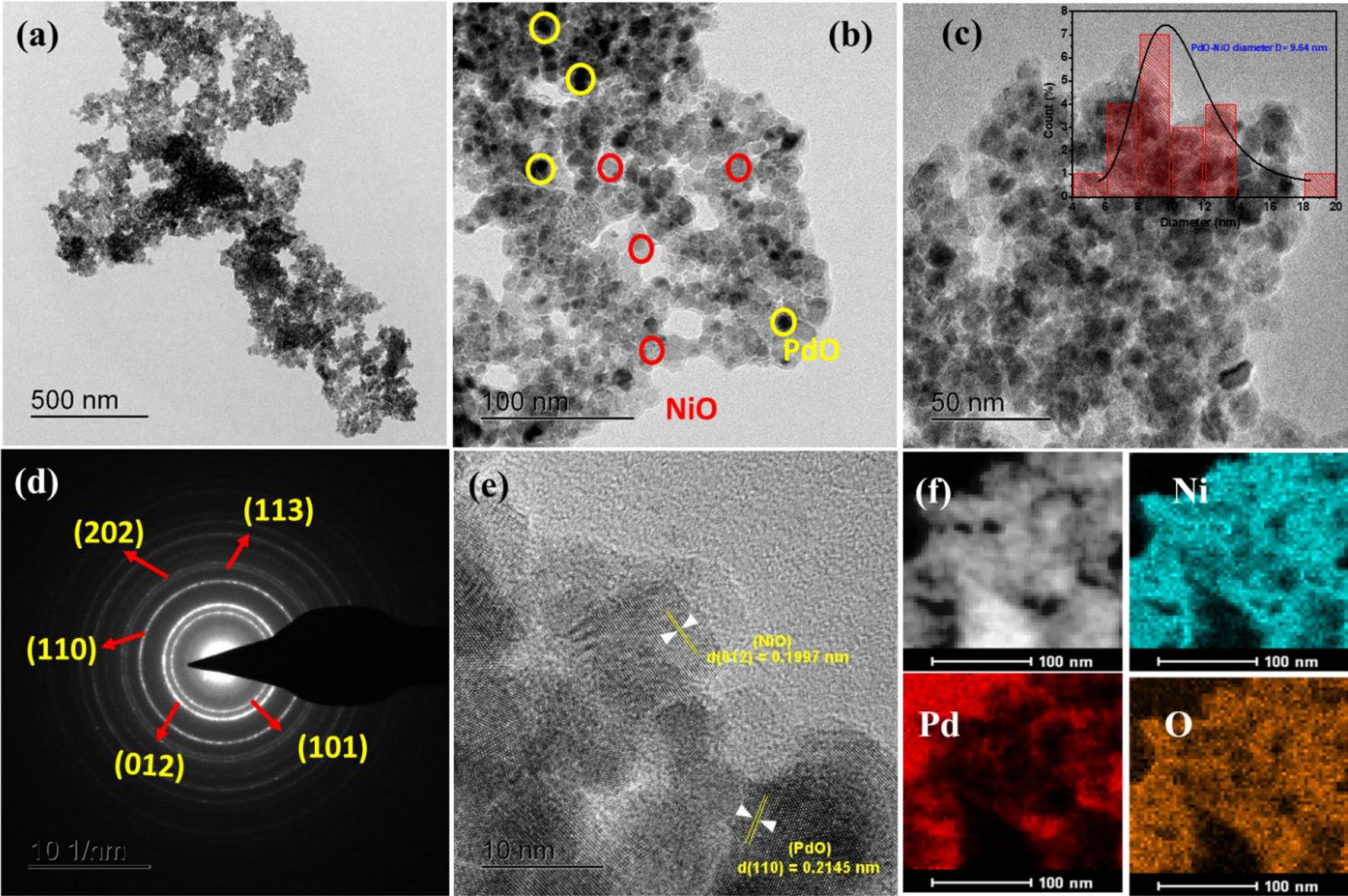
High resolution transmission electron microscopy HRTEM images (**a**–**c**), (**d**) SAED pattern, (**e**) interplanr spacing and (**f**) elemental mapping of PdO–NiO nanocomposite.

The detailed morphology and particle size distribution of the PdO–NiO NPs was measured by HR-TEM and the results are presented in Fig. 4. Figure 4a–c shows the typical HRTEM images of the as-synthesized PdO–NiO nanocomposite. The obtained TEM images confirmed the uniform distribution of the spherical PdO–NiO NPs, which agrees with FESEM results. In Fig. 4c (inset), the histogram reveals that formed PdO–NiO nanoparticles are uniformly distributed with an average particle size of about 9.64 ± 2.1 nm, which is well associated with XRD crystallite size. Furthermore, the SAED pattern was analyzed to understand the crystallinity and the crystal quality of the PdO–NiO nanoparticles are shown in Fig. 4d. The clear ring-like structure suggests the polycrystalline nature of PdO–NiO. The obtained diffraction rings d-spacing values are corresponding to the (101), (012), (110), (113) and (202) planes of the NiO nanoparticles. Figure 4e shows the lattice fringes of the PdO doped NiO nanoparticles. The fringes show the lattice planes for both metal oxides. The interplanar d-spacing value of 0.1997 nm to correspond to the (012) plane of the NiO phase and the d-spacing value of 0. 2145 nm to correspond to the (110) plane of the PdO in the composite. Which is well correlated with the XRD d-spacing values. Elemental mapping in Fig. 4f shows the presence of Ni, Pd and O elements with uniform distribution as similar as SEM mapping. The morphology results of the synthesized catalysts are well associated with XRD, FTIR and XPS analysis.

**Figure 5:**
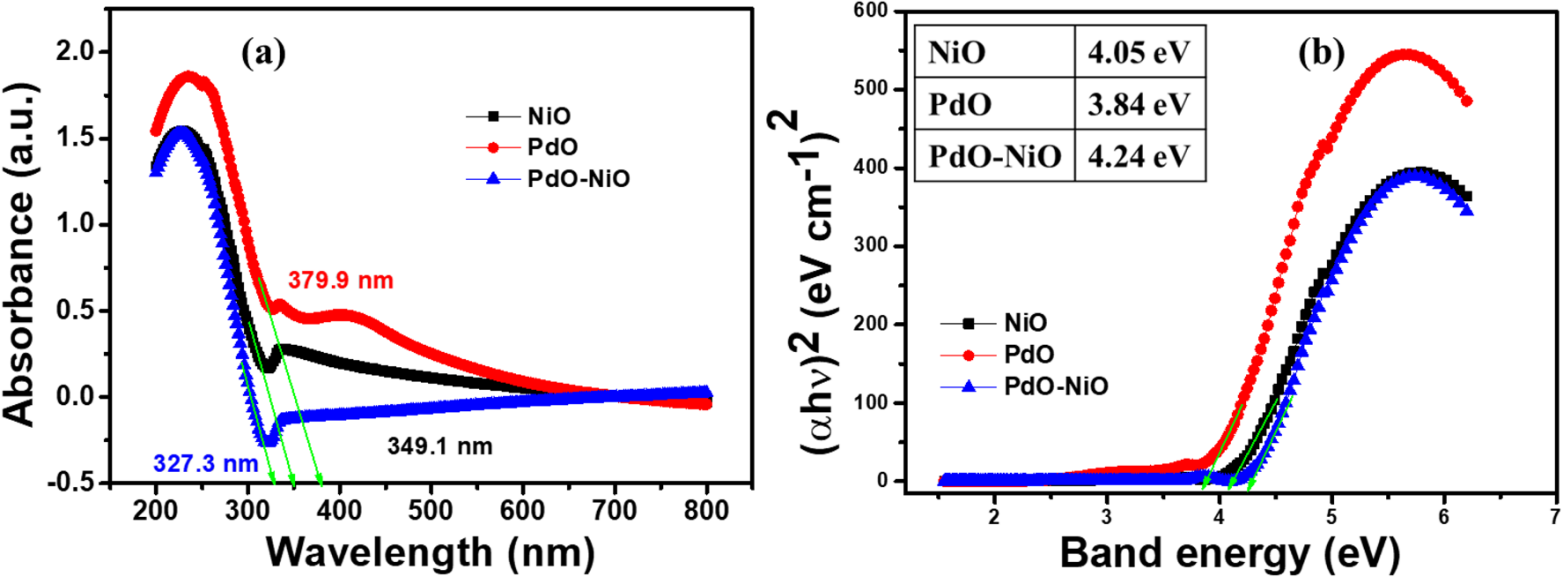
(**a**) UV–Vis absorption spectra and (**b**) Plot of (αhν)^2^ Vs hν for NiO, PdO and PdO–NiO nanocomposite (Inset shows the bandgap energy of the catalyst).

UV–Vis absorption spectra were analyzed for the as-synthesized catalysts NiO, PdO and PdO–NiO and the respective results are presented in Fig. 5a. The absorption spectra show the strongest absorption maxima at 234.8 nm for all three catalysts. In addition, the characteristic absorption band of NiO and PdO were observed at 338.2 nm and 422.1 nm respectively^23^, on the other hand, no characteristic absorption band was observed for PdO–NiO sample. Furthermore, the bandgap energy was calculated for three catalysts by using the Schuster-Kubelka–Munk function.

1 $$(\alpha {\text{h}}\nu )\,{\text{n}} = {\text{A}}({\text{h}}\nu - {\text{Eg}})$$

The bandgap energy (E_g_) was achieved by extrapolating against the photon energy and the obtained results are shown in Fig. 5b. The calculated bandgap (E_g_) of NiO, PdO and PdO–NiO are 4.05 eV, 3.84 eV and 4.24 eV, respectively^25^ (Fig. 5b, inset). The PdO doping with NiO increases its bandgap value. This suggests that the PdO interface and NiO interface are closely combined in the composite. The obtained band gap value of the catalysts is much higher than the reported bandgap energy. The bandgap energy is highly dependent on the particle's size. The bandgap energy increases with decreasing particle size, which confirmed that the synthesized catalysts are in nanoscale. The bandgap energy (Eg) of the PdO–NiO catalyst is well associated with FESEM and TEM results.

**Figure 6:**
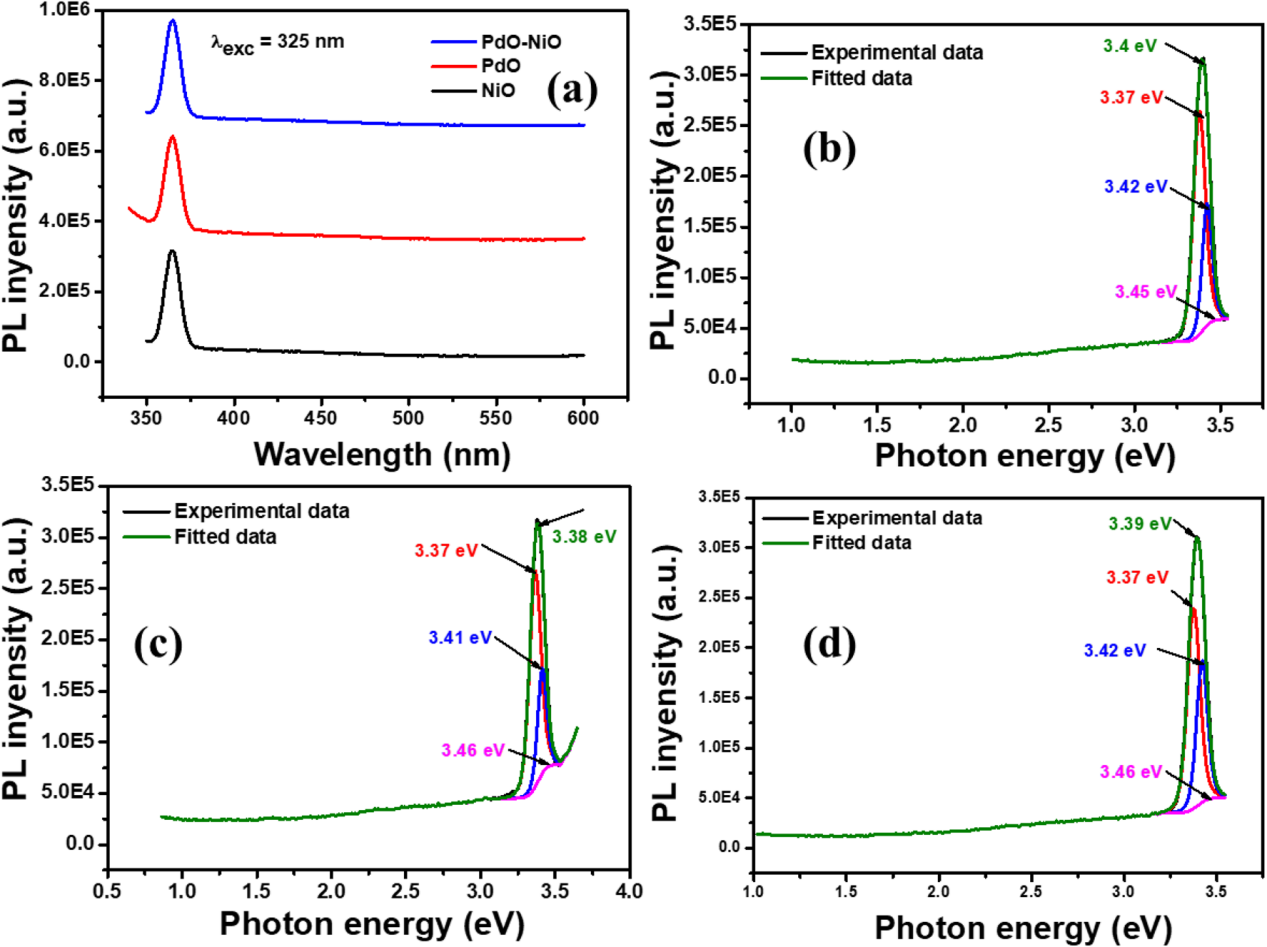
(**a**) PL spectra and (**b**–**d**) deconvoluted spectra of NiO, PdO and PdO–NiO nanocomposite respectively.

The photoluminescence (Pl) spectra of NiO, PdO and PdO–NiO materials were measured at 325 nm excitation wavelength and presented in Fig. 6. Figure 6a shows the Pl spectra of the pure NiO, PdO and PdO–NiO nanocomposite. The blue/violet emission was observed for all three samples at 364 nm due to the excitation of 3d^8^ electrons of Ni^2+^ ions from the conduction band to the valence band^24^. From Fig. 6a, it can be seen that the intensity of the PdO–NiO nanocomposite is lower than the pure NiO and PdO, which indicated the higher electron transfer between the NiO and PdO, which is well correlated with electrochemical results. The deconvoluted PL spectra of NiO, PdO and PdO–NiO materials are shown in Fig. 6b–d; four peaks have been fitted for each sample as shown in Fig. 6b–d. The UV emission at 364 nm (3.4 eV) corresponds to the near band edge (NBE) excitation of NiO^25^. The obtained PL spectra confirmed that the PdO–NiO nanocomposite has more conductivity than the pure metal oxides.

**Figure 7:**
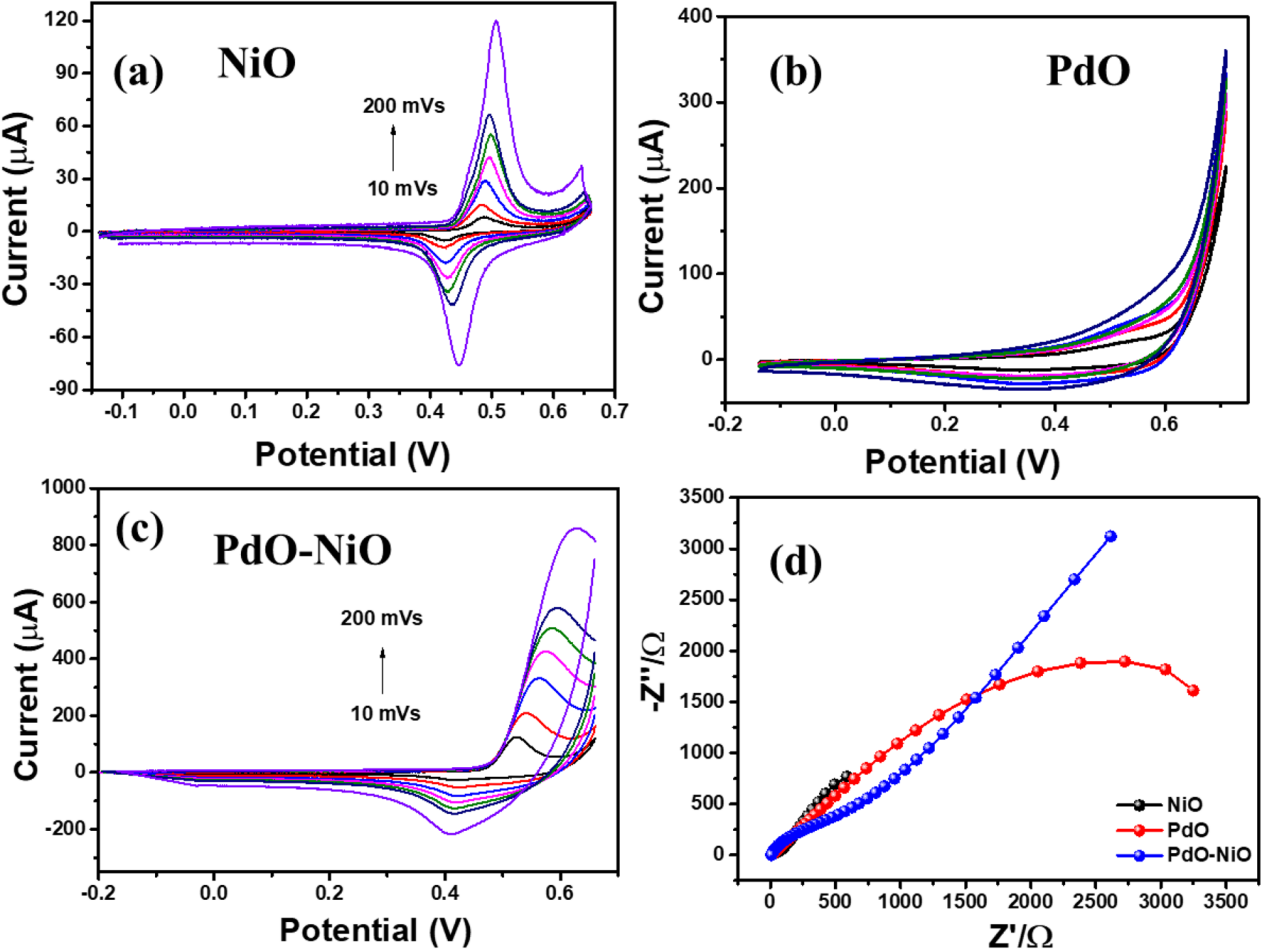
(**a**–**c**) Cyclic voltammetry and (**d**) EIS curves of NiO, PdO and PdO–NiO nanocomposite in 1 M KOH electrolyte solution.

The electrode kinetics of the NiO, PdO and PdO–NiO modified GC electrode was explored in 1 M KOH at different scan rate variation at room temperature. In addition, the resistance of the aforesaid electrodes was monitored in impedance analysis and shown in Fig. 7. Figure 7a, the NiO/GC show a pair of well-defined redox peaks at around 0.49 V and 0.44 V respectively, which corresponding to the reversible reaction between Ni^2+^ and Ni^3+^^26^. In addition, the redox peak currents linearly increase with increasing scan rate.

Figure 7b shows CV pattern of PdO/GC electrode in 1 M KOH solution, which sows poor peaks palladium oxide and palladium reduction peak at 0.52 V and 0.53 V respectively due to the formation of oxyhydroxide on the electrode surface in basic medium. Whereas the PdO–NiO NPs modified GC electrode in Fig. 7c show well-defined Ni^2+^ and Ni^3+^ kinetics with eightfold higher peak current. Which confirms the Efficient electron transfer between NiO and PdO in the composite. Which is well associated with PL results. Furthermore, the impedance spectra were achieved for three electrodes at fixed over potential (500 mV s^−1^) in 1 M KOH electrolyte and presented in Fig. 7d. In Fig. 7d the Rct value of the pure metal oxides NiO/GC and PdO/GC electrode were obtained as 236 Ω and 2702 Ω respectively. Whereas the bimetal oxide PdO–NiO/GC show 425.7 Ω, which is lower than the pure PdO. Due to the superior electron transformation between each metal oxide.

### Catalytic reduction of Azo compounds

**Figure 8:**
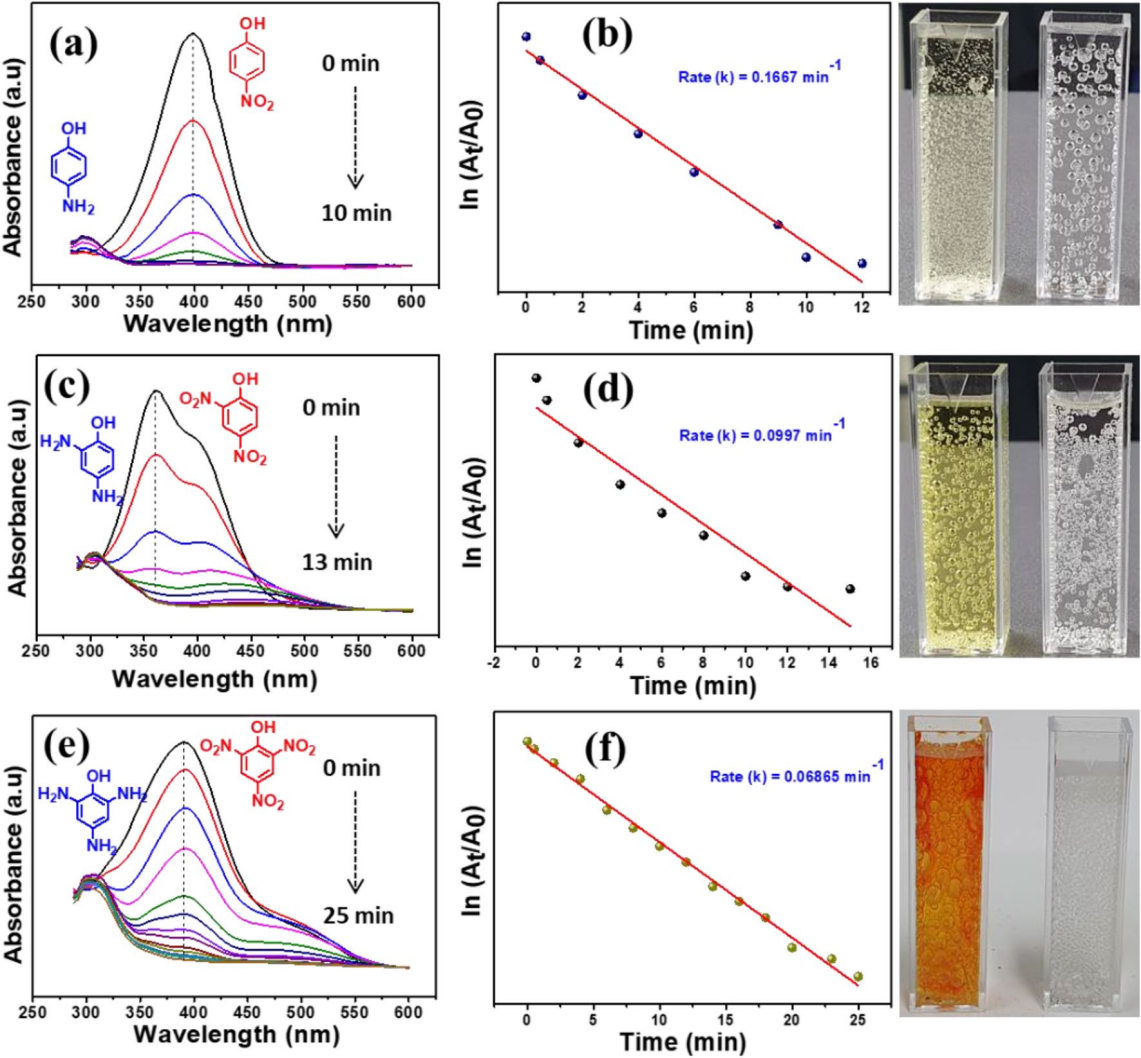
Catalytic activity and kinetic rate of PdO–NiO nanocomposite on reduction of nitrophenols with NaBH_4_ solution (**a**,**b**) NP, (**c**,**d**) DNP and (**e**,**f**) TNP.

Azo compounds are highly toxic to the environment as well as human beings. Especially, nitrophenols are listed as the topmost hazardous chemical in the world. Hence the reduction of nitrophenols gains the most attention. Generally, the nitrophenol reduction reaction is thermodynamically favourable (E^0^ = − 0.76 V) at optimized conditions, whereas the NaBH_4_ acts as a reducing agent (E^0^ = − 1.33 V)^27,28^. However, the reduction rate is prolonged without the catalyst due to the kinetic barrier between the reducing agent and reactant. Hence, the catalytic reduction nitrophenols are a good way to convert to non-toxic aminophenol (AP) with the presence of NaBH_4_ as a reducing agent. The reduction reaction was easily monitored with a UV–Vis spectrometer. It is known that NaBH_4_ alone cannot reduce the nitrophenols into aminophenol’s. As shown in Fig. SI. 2 the fresh nitrophenols (NP, DNP and TNP) absorption peak appeared at 300–370 nm respectively. When the addition of reducing agent, the peak was shifted to 402–450 nm^28^. In addition, the solution color was turned light yellow to deep yellow, due to the formation of corresponding nitrophenolate ions in basic solution. However, no reduction was achieved over 2 h, indicating that nitrophenolate ions were very stable with NaBH_4_. Furthermore, the catalytic activity of the NiO was explored with three nitrophenols and shown in Fig. SI. 3. The pure NiO exhibits a poor catalytic reduction of nitro compounds. In contrast, the PdO–NiO catalyst show excellent activity on the nitrophenols, as shown in Fig. 8.

It can be seen that the nitrophenolate peak absorbance at 400 nm gradually decreases with reaction time, which confirmed that PdO–NiO promotes the electron and hydrogen transfer between the reactant. Due to the higher active sites of the PdO–NiO. The present PdO–NiO catalyst completes the reduction reaction of NP, DNP and TNP within 10, 13, 25 min respectively. In addition, the aminophenol absorption peak appeared around 300 nm for all three nitrophenols. On the other hand, the deep yellow solution turned colorless, indicating the formation of aminophenol. The rate constant κ_app_ for each nitrophenol was calculated from the plot of ln (A_t_/A_o_) Vs. time. The proposed PdO–NiO catalyst exhibits excellent rate constant 0.1667, 0.0997, 0.0686 min^−1^ for NP, DNP and TNT, respectively, which is the higher rate constant than the previously reported catalyst (Table SI. 2). Generally, the reduction mechanism of nitrophenols to aminophenols follows many intermediate steps from nitro to nitroso and then to hydroxylamine and to final aminophenol. For these reaction required both electron transfer and active hydrogen atoms. Here, the BH_4_^−^ ions produce the active hydrogen atoms on the surface of the catalyst and subsequently, the PdO–NiO catalyst enhances the electron transfer. As a result, the reduction of NP could be efficiently accelerated by the PdO–NiO catalyst. Furthermore, comparison of the catalytic reduction performance of nitrophenols with varies catalyst are shown in Table SI. 2. The reduction mechanism of the nitrophenols with PdO–NiO catalyst with NaBH_4_ as shown in Fig. SI. 6.

**Figure 9:**
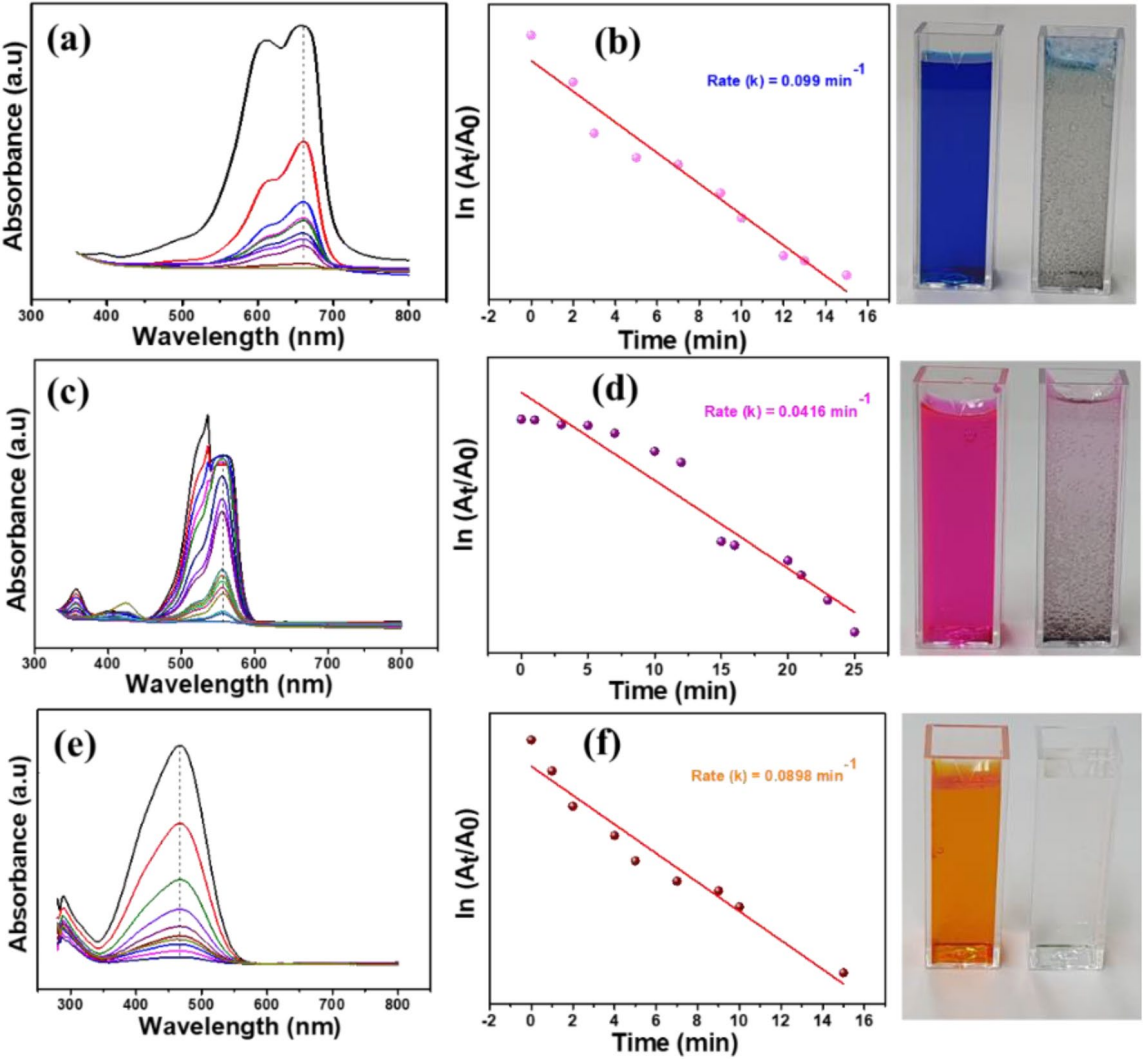
Catalytic activity and kinetic rate of PdO–NiO nanocomposite on reduction of azo dyes with NaBH_4_ solution (**a**,**b**) MB, (**c**,**d**) RhB and (**e**,**f**) MO.

**Figure 10:**
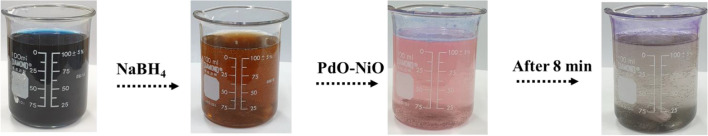
Reaction progress of an azo compounds mixture (4-NP, 2,4-DNP, 2,4,6-TNP, MB, RhB, MO) with PdO–NiO and NaBH_4_. Conditions: Dye: 100 ppm, 25 ml, Nitrophenol: 0.12 mM, 25 ml, NaBH_4_: 0.1 M, 5 ml and catalyst: 3 mg.

Furthermore, the catalytic activity of PdO–NiO composite was explored by the reduction of organic azo dye compounds such as Methylene blue (MB), Rhodamine B (RhB) and Methyl orange (MO) with the addition of NaBH_4_ in the presence of PdO–NiO catalyst^29^. The reduction of each dye was monitored at different absorption peaks, as shown in Fig. 9. The intensity of each dye at respective wavelengths linearly reduced with time in the presence of the PdO–NiO. In addition, the reduction rate κapp for each dye was calculated from the plot of ln (A_t_/A_o_) Vs. time. PdO–NiO catalyst exhibited excellent reduction rate as 0.099, 0.0416 and 0.0896 min^−1^ for MB, RhB and MO, respectively, superior catalytic activity than previous reports. In addition, the azo dye solution turned into colourless, indicating the complete reduction occurs in the presence of PdO–NiO. The pure NiO catalyst exhibits poor reduction activity towards azo dyes with the presence of NaBH_4_ (Fig. SI. 4). Furthermore, comparison of the catalytic reduction performance azo dyes with varies catalyst are shown in Table. SI. 3. Additionally, the reduction of the mixture of nitrophenols (NP, DNP and TNP) and azo dyes (MB, RhB and MO) was tested with PdO–NiO nanocomposite and obtained results are shown in Fig. 10. Initially, the mixture of azo compounds is formed dark solution then rapidly turned into a colourless and became a transparent solution with the addition of PdO–NiO in the presence of NaBH_4_^30^. The complete azo compounds reduction was achieved within 8 min. Hence, the proposed PdO–NiO is a promising catalyst for wastewater treatment. In addition, the effect of the catalyst loading on the reduction of mixture of azo compounds were studied with different loading amount of PdO–NiO catalyst (3–10 mg) and shown in the Table. SI. 4. Which show that the reduction rate increased with loading amount of the catalyst. In addition, the catalytic reduction performance of toxic azo compounds by various catalysts are shown in Table. 2. PdO–NiO catalyst exhibit superior reduction performance than the previously reported catalyst.

**Table 2:** Comparison of catalytic reduction performance of toxic azo compounds by various catalysts.

Mixture of azo compounds	Catalysts	[Mixture] (mM)	Volume of soln	[NaBH_4_] (M)	Reaction time (min)	Refs.
20 ml NP + MB + RhB + MO	RGO/Co	40 ppm	80 ml	1.5	15	^31^
10 ml NP + MB + RhB + MO	Co_3_O_4_/HNTS	10 ppm	3 ml	0.1	5	^32^
25 ml of 4-NP + DNP + TNP + MB + RhB + MO	PdO–NiO NPs	(Dye: 100 ppm; NP: 20 ppm)	150 ml	0.1	8	This work

Furthermore, the reduction mechanism of azo dyes over PdO–NiO catalyst with reducing agent shown in Fig. SI. 7.

**Figure 11:**
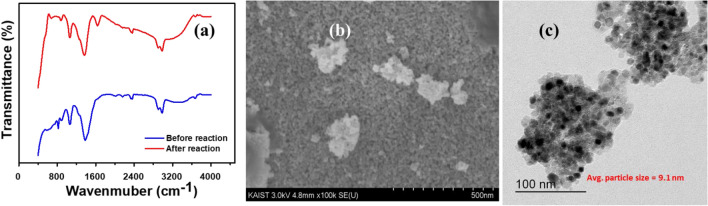
(**a**) FTIR spectra of PdO–NiO nanocomposite before and after reducing the mixture of azo compounds (**b**) FE-SEM image (**c**) HR-TEM image of PdO–NiO after reducing the mixture of azo compounds.

After the complete reduction reaction, the catalyst property was analyzed to understand the stability of PdO–NiO. In Fig. 11a, FTIR spectra showed no noticeable changes before and after catalytic reduction of mixture reduction. Additionally, the SEM image (Fig. 11b) also showed no changes in the morphology of the PdO–NiO. In addition, HR-TEM image (Fig. 11c) was also analyzed to study the change in the particles size after catalytic reduction, show no considerable change in the particle size. Which proved that PdO–NiO is highly stable in the reduction conditions.

## Conclusion

In summary, we have synthesized the low-cost, PdO-doped NiO heteromixture via a simple and cost-effective hydrothermal combined calcination process. The XRD, XPS, FE-SEM and HR-TEM techniques confirmed that, the spherical PdO nanoparticles are evenly doped with NiO nanoparticles. The bi-metal oxide composite PdO–NiO showed high band gap value 4.24 eV. PdO–NiO enhanced the superior catalytic activity over the pure NiO. The proposed PdO–NiO catalyst exhibit excellent rate constant 0.1667, 0.0997, 0.0686 min^−1^ for NP, DNP and TNT and 0.099, 0.0416 and 0.0896 min^−1^, for MB, RhB and MO dyes respectively. Which is the higher rate constant than the previously reported catalyst. Additionally, PdO–NiO completes the reduction of the mixture of azo compounds within 8 min. Further, PdO–NiO exhibits a stable reduction rate of azo compounds over five cycles with no significant loss. Moreover, a plausible mechanism for the catalytic reduction of nitrophenols and azo dyes was proposed. Hence, the proposed low-cost and high efficient PdO–NiO catalyst could be a promising catalyst for the degradation of azo compounds.

## Experimental

### Materials and method

The metal precursors (Ni(NO_3_)_2_·6H_2_O) (< 99.95%), (PdCl_2_) (< 99.99%), (Na_2_CO_3_) (< 99.99%) and (KOH) (extra pure) all chemicals were obtained from Dae-Jung chemicals, Korea. The dyes such as Methylene blue, Rhodamine B, and Methyl orange were purchased from Dae-Jung chemicals, Korea. The Aromatic nitrophenols (NP, DNP, TNP) and NaBH_4_ (< 99.99%) were purchased from Sigma Aldrich, Korea.

### Catalyst preparation

The bimetal oxide (PdO–NiO) was synthesized by simple co-precipitation method. Firstly, 0.443 g PdCl_2_ and 8.36 g Ni(NO_3_)_2_·6H_2_O were dissolved in 25 mL of DI water until it became the homogenous solution. Then the solution pH was increased to 9.5 through a slow addition of Na_2_CO_3_ solution. Then, the green slurry was formed which was rapidly mixed at 60 °C for 5 h. then the precipitate was separated by vacuum filtration and thoroughly washed with DI water until the filtrate solution became pH 7. Then the obtained crystals were dried in an electric oven at 100 °C for 12 h, and then it was finely grained. Finally, the dried-grained sample as calcined at 500 °C for 3 h in the air atmosphere. In a similar manner, the single metal oxide (NiO and PdO) were synthesized only with their respective metal source. A possible reaction mechanism is described for the formation of PdO–NiO, PdO and NiO.

2 $${\text{Ni}}({\text{NO}}_{{3}} )_{{2}} ({\text{aq}}) + {\text{Na}}_{{2}} {\text{CO}}_{{3}} ({\text{aq}}) \, \to {\text{2Na}}({\text{NO}}_{{3}} ) \, ({\text{aq}}) \, + {\text{NiCO}}_{{3}} ({\text{s}}) \, \downarrow$$

3 $${\text{PdCl}}_{{2}} ({\text{aq}}) + {\text{Na}}_{{2}} {\text{CO}}_{{3}} ({\text{aq}}) \, \to {\text{2NaCl}}({\text{aq}}) \, + {\text{PdCO}}_{{3}} ({\text{s}}) \, \downarrow$$

4 $${\text{NiCO}}_{{3}} ({\text{s}}) \, + {\text{PdCO}}_{{3}} ({\text{s}}) \, \mathop{\longrightarrow}\limits^{\Delta }{\text{PdO}} - {\text{NiO}}({\text{s}}) \, + {\text{2CO}}_{{2}} ({\text{g}})$$

### Physical characterizations

The crystalline phase and structure of PdO–NiO, NiO and PdO were obtained from X-ray diffractometer (Bruker AXS company, Germany). Functional groups and metal oxide peaks of the catalysts was analyzed by ATR-FTIR (iS5, Thermo Scientific, USA). Morphology images and elemental composition of metal oxides were monitored by (FE-SEM) Hitachi S-4800 II (Japan). The HR-X-ray &UV Photoelectron Spectrometer (XPS, Thermo scientific, USA) was used to analyze the metal bonding. High resolution images and mapping images of the catalyst are analyzed from HR-TEM (Hitachi) instrument. The PL excitation and emission spectra were obtained by using HORIBA Fluoromax spectrofluorometer. The catalytic reduction of nitrophenol and dye molecules were systematically studied by using UV–Vis spectrophotometer (Shimadzu).

### Electrochemical characterizations

The electrochemical analysis was carried out using a standard three-electrode system. Catalyst (PdO–NiO, NiO, and PdO) modified Glassy carbon electrode (GCE) was used as working electrode, Pt wire and Ag/AgCl were fixed as counter and reference electrode respectively. The electrochemical activity of the catalysts was analyzed in 1 M KOH electrolyte over a range of − 0.2 to 0.9 V. In addition, the electrochemical impedance analysis of the catalysts modified GCE was analyzed in 1 M KOH solution under different amplitude.

### Catalytic activity analysis

Nitrophenol reduction reaction was performed using NaBH_4_ as the reductant in an aqueous medium. In this work, the nitrophenol reduction was performed with three different nitrophenols (2-NP, 2,4-DNP and 2,4,6-TNP) solution with an initial concentration of 0.1 mM. Firstly, 5 ml of freshly made 0.1 M of NaBH_4_ was added into 50 mL of the nitrophenol solution and then 2 mg/ml of PdO–NiO was mixed and then each nitrophenols reduction was monitored at every 1 min respectively. In similarly, the azo dyes (MB, RhB and MO) reduction was analyzed with 50 ppm of 100 ml azo dyes solution and the ʎ max of each dye was monitored at every 1 min time interval.

## Supplementary Information

Supplementary Information.
