# Supplementary material for: Highly efficient and simultaneous catalytic reduction of multiple toxic dyes and nitrophenols waste water using highly active bimetallic PdO–NiO nanocomposite
Source: Sci Rep. 2021 Nov 22;11:22699. doi: 10.1038/s41598-021-01989-7 (PMC8609031; doi:10.1038/s41598-021-01989-7)
Supplement: Supplementary file 1 — Supplementary Information. [file 41598_2021_1989_MOESM1_ESM.docx]

**Highly efficient and simultaneous catalytic reduction of multiple toxic dyes and nitrophenols waste water using highly active bimetallic PdO-NiO nanocomposite.**

A.G. Ramu^a^ and Dongjin Choi^a^ *

*^a^ Department of Materials Science and Engineering, Hongik University, 2639-Sejong- ro, Jochiwon- eup, Sejong-city, 30016, Republic of Korea.*

*Corresponding author Email address: [djchoi@hongik.ac.kr](mailto:djchoi@hongik.ac.kr) (Dongjin Choi)

| **Material** | **2θ degrees** | **d-spacing (nm)** | | |
| --- | --- | --- | --- | --- |
|  |  | **(hkl)** | **Theoretical** | **Experimental** |
| **PdO nanoparticles** | | | | |
| PdO | 28.35085 | (100) | 0.3043 | 0.3144 |
| PdO | 33.66539 | (101) | 0.2643 | 0.2659 |
| PdO | 41.75741 | (110) | 0.2152 | 0.2160 |
| PdO | 54.52989 | (112) | 0.1675 | 0.1680 |
| PdO | 60.27939 | (103) | 0.1536 | 0.1533 |
| PdO | 71.05721 | (202) | 0.1322 | 0.1325 |
| **NiO nanoparticles** | | | | |
| NiO | 37.16952 | (101) | 0.2410 | 0.2416 |
| NiO | 43.24615 | (012) | 0.2088 | 0.2089 |
| NiO | 62.81731 | (110) | 0.1476 | 0.1477 |
| NiO | 75.3273 | (113) | 0.1259 | 0.1260 |
| NiO | 79.34261 | (202) | 0.1205 | 0.1206 |
| **PdO-NiO nanoparticles** | | | | |
| PdO-NiO | 34.27637 | (002) | - | 0.2613 |
| PdO-NiO | 37.20791 | (101) | - | 0.2413 |
| PdO-NiO | 43.26261 | (012) | - | 0.2088 |
| PdO-NiO | 62.81864 | (110) | - | 0.1477 |
| PdO-NiO | 75.32305 | (113) | - | 0.1260 |
| PdO-NiO | 79.3379 | (202) | - | 0.1206 |

**Table SI. 1** d-spacing values of the PdO, NiO, and PdO-NiO nanocomposite.


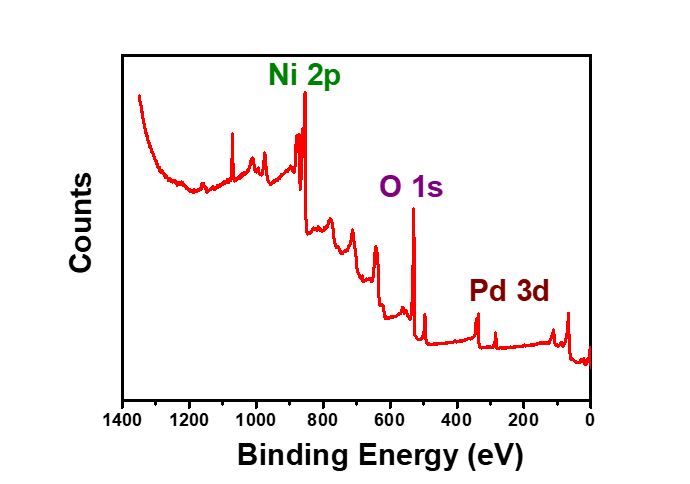


**Fig. SI. 1** Wide scan XPS spectrum of the PdO-NiO.


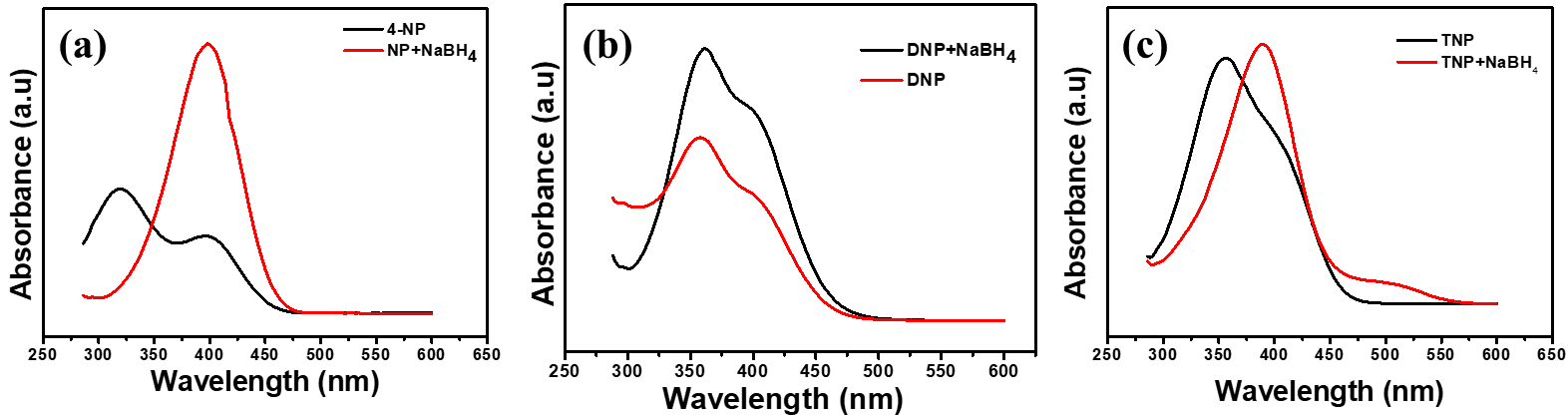


**Fig. SI. 2** UV absorption spectra of (a) 4-NP, (b) DNP and (c) TNP with NaBH_4_.


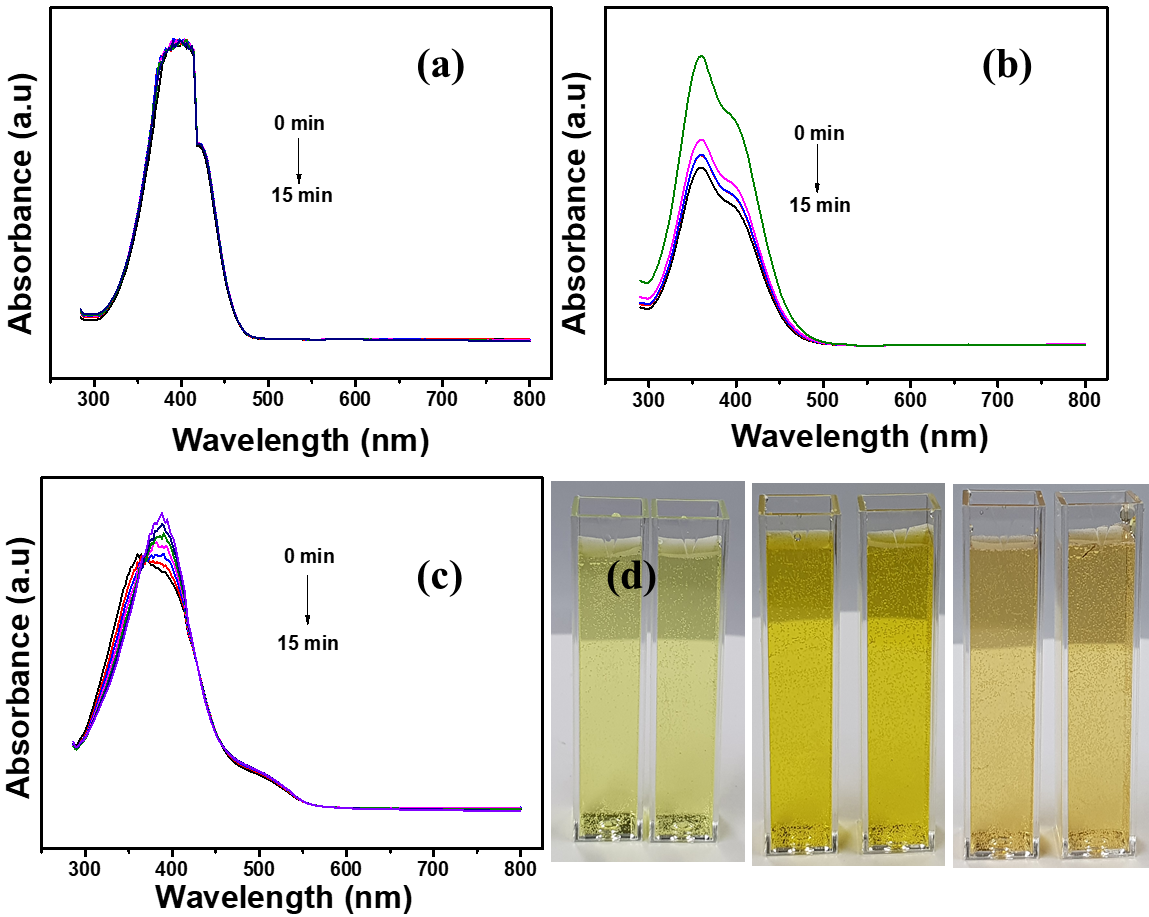


**Fig. SI. 3** Catalytic reduction of (a) 4-NP, (b) DNP and (c) TNP with NaBH_4_ in the presence of NiO.


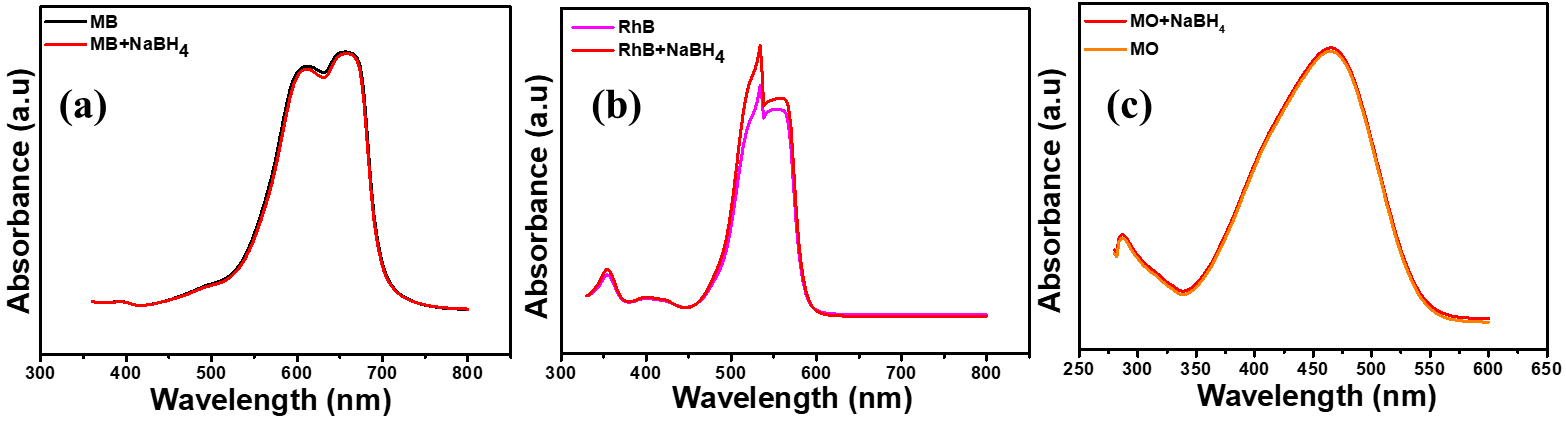


**Fig. SI. 4** UV absorption spectra of (a) MB, (b) RhB and (c) MO with NaBH_4_.


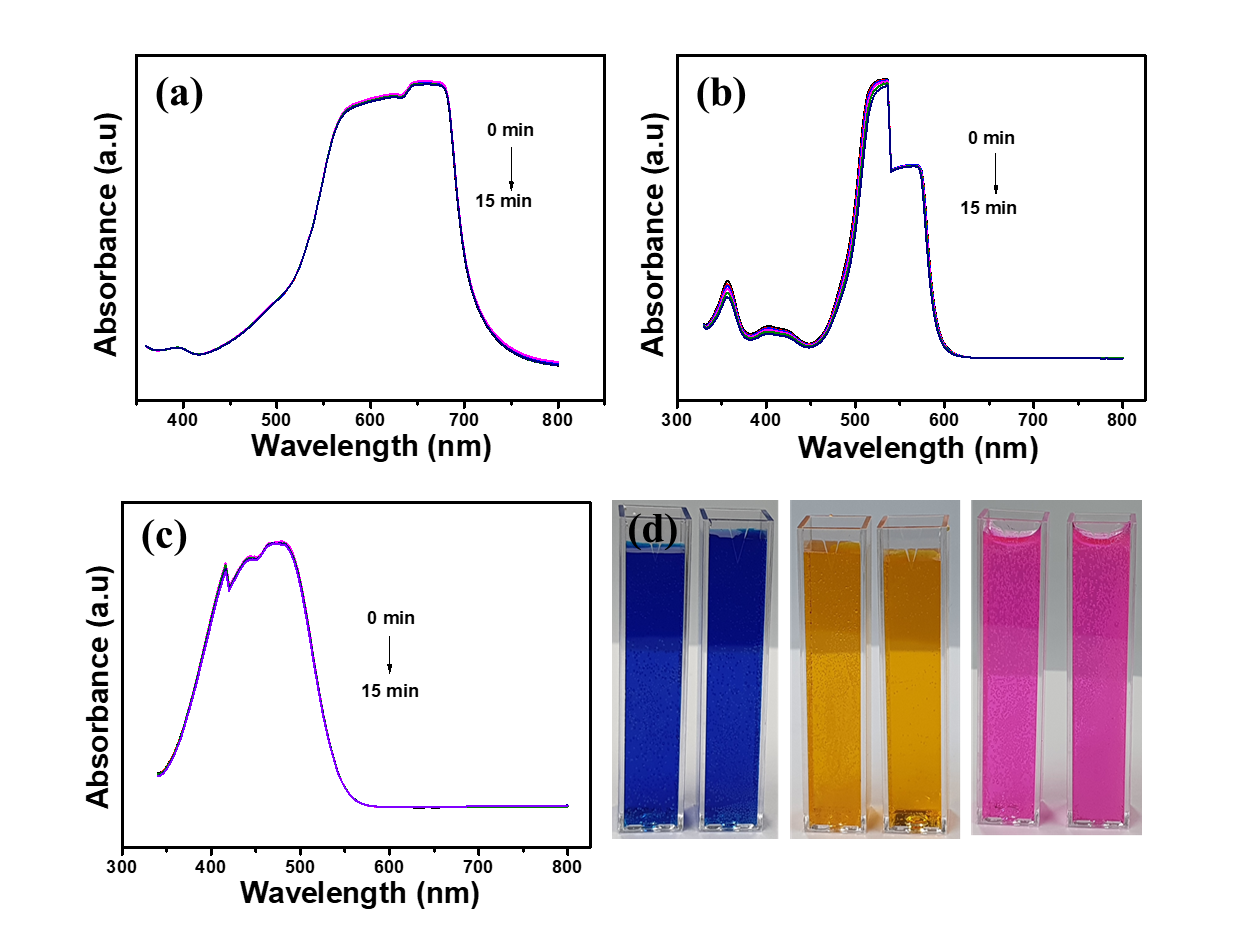


**Fig. SI. 5** Catalytic reduction of (a) MB, (b) RhB and (c) MO with NaBH_4_ in the presence of NiO.

**Table. SI. 2** Comparison of catalytic performance for reduction of variety of nitrophenols by NaBH_4_ in the presence of different catalysts

|  | **Catalysts** | **[NP]**  **(mM)** | **Volume of dye** | **[NaBH_4_] (M)** | **Reaction**  **Time**  **(min)** | **Rate constant**  ***K_app_* (min^-1^)** | **Refs.** |
| --- | --- | --- | --- | --- | --- | --- | --- |
| **4-NP** | Fe/CC-CH | 0.1 | 3 ml | 0.1 | 18 | 0.2937 | [8] |
|  | Au/CA | 0.1 | 2.5 ml | 0.1 | 45 | 0.1-0.20 | [9] |
|  | Ag/CB-CH | 0.1 | 2.5 ml | 0.1 | 6 | 0.1040 | [10] |
|  | **PdO-NiO NPs** | **0.1** | **50 ml** | **0.1** | **10** | **0.1667** | **This work** |
| **DNP** | AuNPs | 10 | 900 | 0.1 | 36 | - | [11] |
|  | Ag@Acl NPs | 6 | 2 | 0.1 | 60 | 0.052 | [12] |
|  | AgNP-PSAC | 0.2 | 5 | 0.7 | 22 | - | [13] |
|  | **PdO-NiO NPs** | **0.1** | **50 ml** | **0.1** | **13** | **0.0997** | **This work** |
| **TNP** | ZnO nanorod | 6 | 2 ml | 0.1 | 18 | 0.141 | [14] |
|  | AuNPS | 0.1 | 1.7 ml | 0.12 | 15 | 0.0026 | [15] |
|  | AgNP-PSAC | 0.2 | 5 | 0.7 | 25 | 0.234 | [13] |
|  | **PdO-NiO NPs** | **0.1** | **50 ml** | **0.1** | **25** | **0.0686** | **This work** |

**Table. SI. 3** Comparison of catalytic performance for reduction of variety of dyes by NaBH_4_ in the presence of different catalysts.

|  | **Catalysts** | **[dye]**  **(M)** | **Volume of dye** | **[NaBH_4_] (mM)** | | **Reaction**  **Time**  **(min)** | **Rate constant**  ***K_app_* (min^-1^)** | **Refs.** |
| --- | --- | --- | --- | --- | --- | --- | --- | --- |
| **RhB** | Fe_3_O_4_/Ag | 1.045×10^-5^ | 25 ml | | 50 | 15 | 0.42 | [1] |
|  | Fe_3_O4@PANI@Au | 2.5×10^-5^ | 20 ml | | 10 | 18 | - | [2] |
|  | SiNWAs-Cu | 5×10^-5^ | 25 ml | | 10.57 | 14 | 0.0037 | [3] |
|  | Au-PANI | 5×10^-5^ | 10 ml | | 2.5 | 15 | - | [4] |
|  | Co_3_O_4_/HNTs | 2.09×10^-5^ | 2.5 ml | | 25.08 | 2 | 1.687 | [5] |
|  | **PdO-NiO NPs** | **1.04×10^-4^** | **100 ml** | | **10** | **25** | **0.0416** | **This work** |
| **MO** | Ag/TiO_2_ | 1.5×10^-5^ | 25 ml | | 2.65 | 9 | - | [6] |
|  | Cu | 3.0×10^-5^ | 25 ml | | 5.3 | 18.6 | - | [7] |
|  | Fe_3_O_4_/eggshell | 3.0×10^-5^ | 25 ml | | 5.3 | 13 | - | [7] |
|  | Cu/Fe_3_O_4_/eggshell | 3.0×10^-5^ | 25 ml | | 5.3 | 17 | - | [7] |
|  | Co_3_O_4_/HNTs | 3.0×10^-5^ | 2.5 ml | | 36 | 3 | 1.425 | [5] |
|  | **PdO-NiO NPs** | **1.52×10^-4^** | **100 ml** | | **10** | **15** | **0.0898** | **This work** |
| **MB** | Cu/Fe_3_O_4_/eggshell | 3.1×10^-5^ | 25 ml | | 5.3 | 30 | - | [6] |
|  | SiNW As-Cu | 5×10^-5^ | 25 ml | | 10.57 | 10 | 0.0052 | [3] |
|  | Co_3_O_4_/HNTs | 3.1×10^-5^ | 2.5 ml | | 37.20 | 10 | 0.155 | [5] |
|  | **PdO-NiO NPs** | **1.56×10^-4^** | **100 ml** | | **10** | **15** | **0.099** | **This work** |

**Table SI. 4** the catalytic activity for the reduction of azo compounds mixture by PdO-NiO with NaBH_4_.

| **PdO-NiO** | **3 mg** | **5 mg** | **8 mg** | **10 mg** |
| --- | --- | --- | --- | --- |
| **Reduction time**  **(min)** | **8** | **7.3** | **6** | **5.2** |


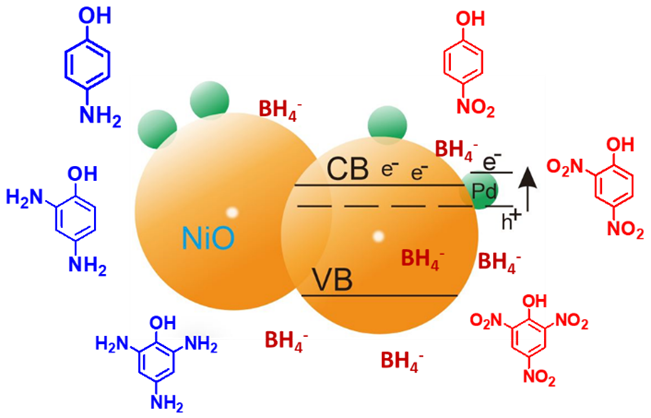


**Fig. SI. 6** Reduction mechanism of nitrophenols over PdO-NiO catalyst with NaBH_4_.


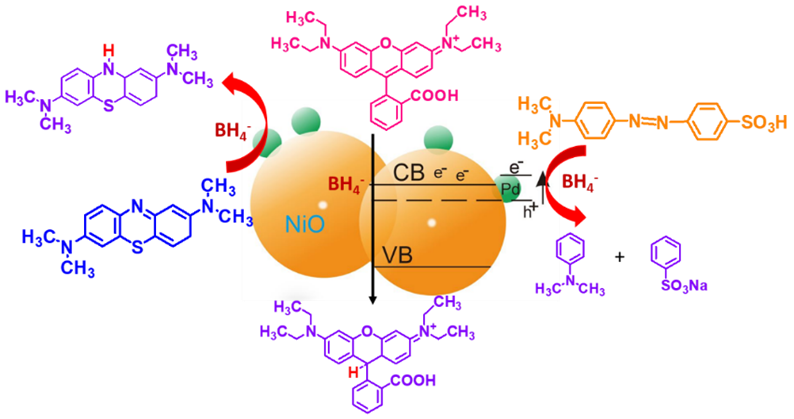


**Fig. SI. 7** Reduction mechanism of azo dyes over PdO-NiO catalyst with reducing agent.

**Reference:**

1. Ai, Lunhong, Chunmei Zeng, and Qinmin Wang. "One-step solvothermal synthesis of Ag-Fe_3_O_4_ composite as a magnetically recyclable catalyst for reduction of Rhodamine B. Catal. Commun. 14, 68-73 (2011).
2. Xuan, Shouhu, Yi-Xiang J. Wang, Jimmy C. Yu, and Ken Cham-Fai Leung. Preparation, characterization, and catalytic activity of core/shell Fe_3_O_4_@ polyaniline@ Au nanocomposites. Langmuir 25, 11835-11843 (2009).
3. Yang, Xiaoling, Hua Zhong, Yihua Zhu, Hongliang Jiang, Jianhua Shen, Jianfei Huang, and Chunzhong Li. "Highly efficient reusable catalyst based on silicon nanowire arrays decorated with copper nanoparticles. J. Mater. Chem. 2, 9040-9047 (2014).
4. Zhang, Bin, Botao Zhao, Shenghui Huang, Ruiying Zhang, Ping Xu, and Hsing-Lin Wang.One-pot interfacial synthesis of Au nanoparticles and Au–polyaniline nanocomposites for catalytic applications. CrystEngComm 14, 1542-1544 (2012).
5. Zhang, Min, Xintai Su, Lida Ma, Aslam Khan, Lu Wang, Jide Wang, A. S. Maloletnev, and Chao Yang. Promotion effects of halloysite nanotubes on catalytic activity of Co_3_O_4_ nanoparticles toward reduction of 4-nitrophenol and organic dyes. J. Hazard. Mater. 403, 123870 (2021).
6. Atarod, Monireh, Mahmoud Nasrollahzadeh, and S. Mohammad Sajadi. Euphorbia heterophylla leaf extract mediated green synthesis of Ag/TiO_2_ nanocomposite and investigation of its excellent catalytic activity for reduction of variety of dyes in water. J. Colloid Interface Sci. 462, 272-279 (2016).
7. Nasrollahzadeh, Mahmoud, S. Mohammad Sajadi, and Arezo Hatamifard. Waste chicken eggshell as a natural valuable resource and environmentally benign support for biosynthesis of catalytically active Cu/eggshell, Fe_3_O_4_/eggshell and Cu/Fe_3_O_4_/eggshell nanocomposites. Appl. Catal. B. 191, 209-227 (2016).
8. Ali, Fayaz, Sher Bahadar Khan, Tahseen Kamal, Khalid A. Alamry, Abdullah M. Asiri, and Tariq RA Sobahi. Chitosan coated cotton cloth supported zero-valent nanoparticles: simple but economically viable, efficient and easily retrievable catalysts. Sci. Rep. 7, 1-16 (2017).
9. Saha, Sandip, Anjali Pal, Subrata Kundu, Soumen Basu, and Tarasankar Pal. Photochemical green synthesis of calcium-alginate-stabilized Ag and Au nanoparticles and their catalytic application to 4-nitrophenol reduction.  Langmuir 26, 2885-2893 (2010).
10. Ali, Fayaz, Sher Bahadar Khan, Tahseen Kamal, Yasir Anwar, Khalid A. Alamry, and Abdullah M. Asiri. Bactericidal and catalytic performance of green nanocomposite based-on chitosan/carbon black fiber supported monometallic and bimetallic nanoparticles. Chemosphere 188, 588-598 (2017).
11. Gerelbaatar, Khongorzul, Ariunzaya Tsogoo, Rentsenmyadag Dashzeveg, Ninjbadgar Tsedev, and Erdene Ochir Ganbold. "Reduction of 2, 4-dinitrophenol to 2, 4-diaminophenol using AuNPs and AgNPs as catalyst. In Solid State Phenomena, vol. 271, pp. 76-84. Trans Tech Publications Ltd, (2018).
12. Devi, Th Babita, Md Ahmaruzzaman, and Shamima Begum. A rapid, facile and green synthesis of Ag@ AgCl nanoparticles for the effective reduction of 2, 4-dinitrophenyl hydrazine. New J Chem 40, 1497-1506 (2016).
13. Sudhakar, Padmaja, and Harnish Soni. Catalytic reduction of Nitrophenols using silver nanoparticles-supported activated carbon derived from agro-waste. J. Environ. Chem. Eng. 6, 28-36 (2018).
14. Bhattacharjee, Archita, and M. Ahmaruzzaman. "A facile and green strategy for the synthesis of 1-dimensional luminescent ZnO nanorods and their reduction behavior for aromatic nitro-compounds. RSC Adv. 6, 527-533 (2016).
15. Wu, Xiao-Qiong, Xing-Wen Wu, Qing Huang, Jiang-Shan Shen, and Hong-Wu Zhang. In situ synthesized gold nanoparticles in hydrogels for catalytic reduction of nitroaromatic compounds. Appl. Surf. Sci. 331, 210-218 (2015).
